# Buprenorphine unobserved “home” induction: a survey of Ontario’s addiction physicians

**DOI:** 10.1186/s13722-019-0146-4

**Published:** 2019-05-01

**Authors:** Anita Srivastava, Meldon Kahan, Pamela Leece, Alison McAndrew

**Affiliations:** Women’s College Hospital, 76 Grenville St., Toronto, ON M5S 1B2 Canada

**Keywords:** Buprenorphine, Physician survey, Home induction, Unobserved

## Abstract

**Background:**

Ontario patients on opioid agonist treatment (OAT) are often prescribed methadone instead of buprenorphine, despite the latter’s superior safety profile. Ontario OAT providers were surveyed to better understand their attitudes towards buprenorphine and potential barriers to its use, including the induction process.

**Methods:**

We used a convenience sample from an annual provincial conference to which Ontario physicians who are involved with OAT are invited.

**Results:**

Based on 85 survey respondents (out of 215 attendees), only 4% of Ontario addiction physicians involved in OAT routinely used unobserved “home” buprenorphine induction: 59% of physicians felt that unobserved induction was risky because it was against “the guidelines” and 66% and 61% respectively believed that unobserved “home” induction increased the risk of diversion and of precipitated withdrawal.

**Conclusions:**

Ontario addiction physicians largely report following the traditional method of bringing in patients for observed in-office buprenorphine induction: they expressed fear of precipitated withdrawal, diversion, and going against clinical guidelines. The hesitance in using unobserved induction may explain, in part, Ontario’s reliance on methadone.

## Background

Buprenorphine/naloxone is an effective treatment for opioid use disorder (OUD) [1] and is now considered a first-line treatment for OUD [2, 3] over methadone treatment because of its favourable safety profile and its feasibility in primary care settings. However, the majority of patients currently on opioid agonist treatment (OAT) in Ontario are still prescribed methadone. In 2017, there were 60,758 patients (4.3 per 1000 population) on OAT in Ontario and, of these, 44,375 were on methadone [4]. This is relevant considering that in 2017, methadone was involved in 211/1265 (16.7%) of opioid-related deaths and cited as one of the three opioids most commonly responsible in single-opioid deaths [5, 6].

Various facilitators and barriers have likely contributed to the current prevalence of methadone prescribing compared with buprenorphine in Ontario. Methadone has been available for the treatment of OUD in Canada for decades, whereas buprenorphine was only approved for use in Canada in 2007, and full public drug coverage^1^ for buprenorphine has been in place in Ontario only since 2016. While the rate of buprenorphine prescribing increased from 0.23 to 0.85 per 1000 Ontarians between 2012 and 2016, the rate of methadone prescribing remained robust and increased from 2.0 to 2.6 per 1000 Ontarians in the same period [7]. This finding suggests that there are other potential barriers to buprenorphine being prescribed.

We hypothesized that one of the main barriers for both addiction physicians and primary care physicians to prescribing buprenorphine is the recommendation that the initial doses of buprenorphine be taken in the office under the observation of a clinician. This could play a role in some addiction medicine prescribers’ preference for methadone overall.

Most buprenorphine guidelines have recommended that the initial dose be taken in an observed clinical setting [8, 9] to prevent diversion, buprenorphine toxicity, and precipitated withdrawal. Yet, the logistics of observed office induction are onerous, as physicians or nurses are required to assess the patient several times over a period of a few hours and the initiation process has been described as a barrier to buprenorphine prescribing [10–13]. Recent studies have demonstrated the safety of unobserved induction [14, 15] and that this is becoming a more widely accepted practice in some jurisdictions [16, 17].

We designed a survey of Ontario OAT providers, who are largely primary care physicians with focused addiction practices, to better understand their attitudes towards buprenorphine. In particular, we explored potential barriers to buprenorphine’s use, including attitudes and practices regarding home induction.

## Methods

### Study design

A cross-sectional survey was designed (Appendix A) to elicit a better understanding of the beliefs and practices around buprenorphine prescribing in Ontario’s OAT prescribing community. The questions were developed by the authors with a view to assessing the outcomes of interest as described in more detail below. The questions were developed by author discussion and consensus and were not adapted from another source. The survey was pre-tested among five family practice residents for clarity and time to completion, which ranged from 5 to 10 min. The survey was then administered to five addiction medicine physicians who prescribe both buprenorphine and methadone to determine relevance and clarity of the questions.

### Study sample

This survey was administered to a convenience sample of Ontario OAT providers at the 2016 annual Ontario OAT providers’ conference held in Toronto, Ontario. The conference was co-hosted by the Centre for Addiction and Mental Health (CAMH) and the College of Physicians and Surgeons of Ontario (CPSO). There were 215 conference registrants: attendees are typically family physicians with focused addiction medicine practices and the conference is intended to provide annual education to these focused practice primary care physicians who provide Ontario’s population with OAT. In 2017, there were a total of 536 registered methadone prescribers in Ontario although they are not necessarily all actively prescribing or practicing (personal communication with the data committee, CPSO) [4]. Most physicians regularly prescribing OAT with focused addiction medicine practices in Ontario are registered methadone prescribers: there may be some who prescribe buprenorphine but are not registered methadone prescribers.

### Data collection

At the conference registration table, conference attendees who were practicing physicians were provided with a paper copy of the survey upon registration. A cover letter accompanied the survey explained its purpose and that research ethics board (REB) approval had been obtained from Women’s College Hospital. Contact information for the investigators and the research ethics board was included in the cover letter. As approved by the REB, formal consent was not obtained from participants: consent was implied by the return of the survey. Attendees who chose to participate in the survey were asked to return their completed surveys at the registration desk before conference departure. There was no incentive offered to complete the survey. An address, but no stamped envelope, was provided. All participants returned their survey at the registration desk: one participant mailed back the survey. No identifying data was requested on the surveys.

### Outcomes of interest

Participants were surveyed on their opinions and practices towards buprenorphine and home induction. Likert scales were used to assess attitudes towards home induction, perceived barriers to buprenorphine use, and current buprenorphine induction practices, all of which were of particular interest.

### Statistical analysis

Survey responses (on paper) were entered into an electronic database and analyzed descriptively. Ten surveys were randomly selected and double-entered to ensure accuracy of data entry.

## Results

We received 88 responses to our survey. Three incomplete surveys were excluded from the analysis as the participants did not answer the likert scale questions. The remaining 85 completed surveys represent 39.5% of the total number of 215 attendees at the 2016 conference. It likely represents a greater percentage of the physician attendees as the 215 attendees include allied health professionals and policy makers.

The group of respondents was relatively new to OAT practice: 35% had only been practicing for 1–5 years and 37% had been practicing for 6–15 years (Table 1). The majority of the physicians identified their practices as being urban (58%) or suburban (20%). Almost half of the physicians (48%) prescribed OAT for more than 100 patients and 13% had more than 300 opioid agonist patients in their practice. A significant percentage of physicians (41%) indicated that they had initiated buprenorphine rather than methadone in more than 40% of their new opioid agonist starts in the past year. The survey explored three main approaches to initiating buprenorphine (Table 2). A large majority (74%) of respondents indicated that they usually or always have patients take their initial doses at the clinic; 31% indicated that they usually or always have patients take the first 1–2 doses observed at the clinic and allow the rest of the day’s dose to be taken unobserved. Only 4% indicated that they usually used unobserved induction for the first few doses of buprenorphine. Yet, when asked about the convenience of unobserved induction, 64% respondents agreed or strongly agreed that it was more convenient for patients and 35% agreed or strongly agreed that it was more convenient for physicians (Table 3).

**Table 1:** Physician demographics

	N (%)
How many years have you been prescribing methadone (N = 82)?	
< 1 year	6 (7)
1–5 years	29 (35)
6–10 years	14 (17)
11–15 years	16 (20)
16–20 years	13 (16)
20+ years	4 (5)
Currently, for how many patients do you prescribe either methadone or buprenorphine (N = 82)?	
1–20	7 (9)
21–50	17 (21)
51–100	18 (22)
101–200	18 (22)
201–300	11 (13)
> 300	11 (13)
In the past year, what percentage of new opioid agonist patients did you start on buprenorphine instead of methadone initiation (N = 82)?	
< 10%	12 (15)
10–20%	13 (16)
21–40%	23 (28)
41–60%	18 (22)
61–80%	9 (11)
81–100%	7 (8)

**Table 2:** Physician practice with respect to buprenorphine induction

	N (%)
Practice	
*Patients attends clinic in withdrawal and all of first day’s doses observed at clinic* (N = 81)	
Rarely or never	8 (10)
Sometimes	13 (16)
Usually	13 (16)
Almost always	47 (58)
*Patients attend clinic in withdrawal,* 1–2 *doses observed, take*-*home doses for rest of the day* (N = 80)	
Rarely or never	27 (34)
Sometimes	28 (35)
Usually	12 (15)
Almost always	13 (16)
*Patients given take*-*home doses for* 1–2 *days, instructed to take first dose after onset of withdrawal* (N = 80)	
Rarely or never	63 (79)
Sometimes	10 (12)
Usually	3 (4)
Experience: I end up prescribing methadone because:	
*The patient doesn’t want to be in withdrawal (N *= *81)*	
Rarely or never	17 (21)
Sometimes	38 (47)
Usually	20 (25)
Almost always	6 (7)
*The patient misses the induction appointment or has trouble arriving in withdrawal on induction day* (*N *= *80*)	
Rarely or never	39 (49)
Sometimes	32 (40)
Usually	8 (10)
Almost always	1 (1)
Almost always	4 (5)

**Table 3:** Physician beliefs on buprenorphine

	N (%)
Compared to office induction, home induction is:	
*Risky because it is against the guidelines* (N = 77)	
Strongly agree	15 (19)
Agree	31 (40)
Neutral	19 (25)
Disagree	7 (9)
Strongly disagree	5 (7)
*Increases the risk of adverse events such as precipitated withdrawal* (N = 78)	
Strongly agree	22 (28)
Agree	26 (33)
Neutral	17 (22)
Disagree	10 (13)
Strongly disagree	3 (4)
*Increases the risk of diversion* (N = 78)	
Strongly agree	17 (22)
Agree	34 (44)
Neutral	14 (18)
Disagree	9 (11)
Strongly disagree	4 (5)
Buprenorphine is preferred over methadone for patients who are adolescents or younger patients (N = 85)	
Strongly agree	33 (39)
Agree	37 (44)
Neutral	8 (9)
Disagree	7 (8)
Strongly disagree	0 (0)
Physicians who lack a methadone exemption should prescribe buprenorphine for patients who must travel long distances to attend a methadone clinic (N = 83)	
Strongly agree	17 (21)
Agree	35 (42)
Neutral	15 (18)
Disagree	10 (12)
Strongly disagree	6 (7)
Compared to office induction, home induction is:	
*More convenient for patients* (N = 77)	
Strongly agree	14 (18)
Agree	35 (46)
Neutral	21 (27)
Disagree	4 (5)
Strongly disagree	3 (4)
*More convenient for physicians (*N = 78)	
Strongly agree	10 (13)
Agree	17 (22)
Neutral	27 (34)
Disagree	18 (23)
Strongly disagree	6 (8)
Physicians who lack a methadone exemption should prescribe buprenorphine:	
*For stable patients transferred from an addiction physician* (N = 83)	
Strongly agree	23 (28)
Agree	23 (28)
Neutral	18 (22)
Disagree	11 (13)
Strongly disagree	8 (9)
*For stable patients who must travel long distances to attend a methadone clinic* (N = 83)	
Strongly agree	17 (21)
Agree	35 (42)
Neutral	15 (18)
Disagree	10 (12)
Strongly disagree	6 (7)
*Rarely, if ever, because they lack the knowledge and skill to prescribe safely* (N = 83)	
Strongly agree	7 (8)
Agree	23 (28)
Neutral	16 (19)
Disagree	29 (35)
Strongly disagree	8 (10)

When asked about barriers to buprenorphine induction, 51% of respondents indicated that they usually or sometimes had to prescribe methadone rather than buprenorphine for patients who were unable to come to the clinic in withdrawal. (Table 2).

When asked about risks of unobserved induction, 59% of physicians agreed or strongly agreed that unobserved induction was risky because it was against “the guidelines,” 66% agreed or strongly agreed that it increased the risk of diversion, and 61% agreed or strongly agreed that it increased the risk of precipitated withdrawal.

We explored other areas of interest related to the uptake of buprenorphine prescribing in primary care and the perceptions of addiction physicians related to the possible advantages and disadvantages of buprenorphine in a primary care setting. Thirty-six percent of respondents agreed or strongly agreed that primary care physicians without focused addiction medicine practices should not prescribe buprenorphine. However, only 19% of physicians disagreed or strongly disagreed with the statement that non-addiction physicians should prescribe buprenorphine when travel to a specialized clinic is very difficult for patients (Table 3).

The majority (82%) of respondents agreed or strongly agreed with the statement that buprenorphine was preferred for patients with benzodiazepine or heavy alcohol use, and most (84%) agreed or strongly agreed that buprenorphine was preferred for patients who were working or were not close to a pharmacy. The vast majority of respondents (83%) agreed or strongly agreed with the statement that buprenorphine is  the preferred treatment choice for youth.

## Discussion

Our finding that only 4% of surveyed Ontario addiction physicians involved in OAT routinely used unobserved “home” induction differs from similar studies in other jurisdictions which have found that unobserved induction is widely used: a survey of physicians in Massachusetts [16] found that 43% of patients were given take-home doses on initiation of treatment. A more recent survey [17] of New York providers found that unobserved buprenorphine induction was standard practice amongst 65% of respondents even though a significant number (38%) felt that concern about diversion was a barrier to buprenorphine prescribing. One possible explanation for the differences between Ontario physicians and those in other jurisdictions is that the former can prescribe both methadone and buprenorphine (methadone is only available in specialized clinics in the United States). Thus, the Ontario physician can prescribe methadone if the patient is unwilling or unable to present to the office in withdrawal for buprenorphine induction.

Our survey results support the hypothesis that many patients who were either eligible or intended for buprenorphine treatment ended up on methadone: 25% of respondents said that they usually ended up prescribing methadone because the patient did not want to be in withdrawal, and almost half said this often occurred. More than 50% indicated that they usually or sometimes had to prescribe methadone because patients would miss their induction appointment or had trouble arriving in withdrawal on the scheduled induction day. This may help explain, in part, the high use of methadone prescribing in Ontario despite the greater educational requirements for physicians prescribing methadone.

It is interesting that the Ontario addiction physicians we surveyed largely did not use unobserved home induction even though cohort studies have established the safety of unobserved induction, with a low incidence of adverse events and good treatment retention rates [14, 15]. Concerns were expressed around “going against the guidelines”: in Ontario, physicians who prescribe methadone have clinical guidelines that are often used as the standard of care to which they are held accountable by the province’s medical licensing authority. This may have created a culture in which addiction physicians are reluctant to go outside the parameters of clinical guidelines in prescribing OAT. The first set of clinical guidelines for buprenorphine in Ontario were published in 2011 [8] and were, until recently, the ones most clinicians likely used in their clinical practices. Those guidelines did not endorse unobserved induction. Newer guidelines in Canada and United States [18–20] recommend caution with unobserved induction and recommend that only experienced providers attempt it.

The acceptance of home induction as a mainstream practice could decrease reliance on methadone and expand access to buprenorphine treatment. Physicians could consider home induction for patients who cannot attend the office in withdrawal for planned buprenorphine inductions rather than feeling that their only option was to initiate methadone. Emergency Department (ED) physicians would be able to prescribe buprenorphine to patients not yet in withdrawal. Primary care physicians similarly may be more amenable to prescribe buprenorphine if they do not have to arrange office induction. Aside from being hard to schedule and time consuming, office induction also creates the impression that buprenorphine induction involves considerable risk and may discourage primary care physicians from incorporating buprenorphine treatment into their practices. Our survey results suggest that addiction physicians are open to working with primary care physicians to expand access to OAT: respondents believed that prescribing physicians should be experienced but that if distance was a barrier for patients then their family doctor should provide OAT.

However, family doctors and addiction physicians may be hesitant to routinely use unobserved “home” induction until there are clinical guidelines clearly stating that it is an acceptable alternative to office induction. Clinical guidelines and practices in Ontario continue to represent a barrier to both its adoption into primary care as well as a barrier to treatment entry for many patients who are unable to present in withdrawal to an ambulatory appointment. The existing recommendations could be revised to more clearly state the acceptability of home induction, as the risks of a small limited supply of buprenorphine tablets are minimal.

There were limitations to our survey. The surveyed group was a convenience sample of physicians at the annual opioid primary care provincial conference. While we are able to capture a sizeable portion of active of Ontario methadone providers (almost half of the registered methadone prescribers in Ontario attended the conference and almost 40% of attendees responded, which is likely more than half the attending physicians), we do not have the demographics of Ontario methadone prescribers as a whole to which we can compare our sample. The demographics of our survey respondents indicated that many were relatively new to practice, were starting new patients on buprenorphine, and the majority had practice sizes of fewer than 200 patients, suggesting that we were likely surveying addiction physicians who were already involved in buprenorphine treatment and who were not very high-volume prescribers.

## Conclusions

In our survey of Ontario addiction physicians who prescribe OAT, the majority use observed office induction. Only 4% were routinely performing unobserved “home” buprenorphine inductions. Many participants stated that office induction was a barrier to treatment for patients, but were apprehensive about possible precipitated withdrawal and about not following clinical guidelines that recommend observed induction. Initiatives to promote provider comfort with buprenorphine home induction may help improve access to opioid use disorder treatment.

## Abbreviations

OUD: opioid use disorder
OAT: opioid agonist treatment
CAMH: Centre for Addiction and Mental Health
CPSO: College of Physicians and Surgeons of Ontario
ED: Emergency Department

## Footnotes

1 Ontario Drug Benefits cover drug costs for all Ontario residents under age 25, over age 64, and anyone receiving social assistance, disability support, long-term care, or prescription drug coverage assistance if they spend a large part of their income on prescription medications.
